# Barriers to Implementation of Evidence Based Practice in Zahedan Teaching Hospitals, Iran, 2014

**DOI:** 10.1155/2015/357140

**Published:** 2015-03-18

**Authors:** Mohammad Khammarnia, Mahsa Haj Mohammadi, Zahra Amani, Shahab Rezaeian, Fatemeh Setoodehzadeh

**Affiliations:** ^1^Health Promotion Research Center, Zahedan University of Medical Sciences, P.O. Box 98135, Zahedan, Iran; ^2^Department of Health Management and Economics, School of Public Health, Tehran University of Medical Sciences, P.O. Box 14155-6446, Tehran, Iran

## Abstract

This study aimed to determine the barriers to implementation of EBP among nurses. This cross-sectional study was conducted in Zahedan City, South East of Iran, in 2014. The questionnaire of barriers to implementation of EBP consists of 27 statements which was distributed among 280 nurses. More than half of the participants agreed that 56% and 57% of barriers to implementation of evidence based practice are related to organizational and individual aspects, respectively. Participants identified barriers at organizational level included the lack of human resources (78.3%), lack of internet access at work (72.2%), and heavy workload (70.0%). Barrier at individual level included lack of time to read literature (83.7%), lack of ability to work with computer (68.8%), and insufficient proficiency in English language (62.0%). Age, educational level, job experience, and employment status were associated with organizational barriers to implementation of EBP. At the individual level only education was associated with barriers to implementation of EBP. Barriers to implementation of EBP occur at both individual and organizational levels. The indicator of quality in nursing practice is EBP. Hence, familiarity with EBP is recommended for Iranian nurses. In addition, knowledge of barriers will help health care system and policy makers to provide a culture of EBP.

## 1. Introduction

Delivery of high-quality and consistent services is a big challenge in the health care system nowadays [1]. Evidence based practice (EBP), a problem-solving approach to patient care based on the best available and valid evidence, leads to enhanced quality of care [2], reduced costs [3], and the individual and professional development of nurses and other health workers [4]. Moreover, EBP based on international standards enhances the quality of clinical practice [5]. EBP has been promoted as a way for clients to receive the best level of care [6].

Nurses are the largest group of health care providers and have a key role in ensuring the promotion of health care [7] and delivering better services [4]. EBP is important to the professional development, responsibility, and capabilities of nurses [5], and it has become an important subject in nursing and has integrated into daily practice [8]. In addition, nurses who practice based on the scientific evidence have been able to make better decisions in services delivery [9]. Though nurses generally report positive attitudes and beliefs towards EBP [9–11], previous studies show that nurses were not familiar enough with its principals and they use EBP to a limited extent [9, 12, 13].

Several studies have found that both human and organizational factors are associated with barriers to the use of EBP including lack of time to read literature, heavy workload, lack of staff experienced in EBP, and lack of resources [11, 14, 15]. A current systematic review showed that there are many barriers to the implementation and use of EBP and concluded that identifying barriers is the first step to removing them [16].

Little research has been conducted on EBP beliefs and its use among Iranian nurses. Moreover, the Iranian nursing care system does not provide the incentive for nurses to engage in research [5] and most nurses were not familiar with the concept of EBP [17].

Therefore, the aim of this study was to determine barriers to implementation of evidence based practice in Zahedan teaching hospitals. The results of the study can help managers and policy makers in planning for better use of EBP in nurses and other staff in hospitals.

## 2. Methods

### 2.1. Study Design

This analytical cross-sectional study was conducted in Zahedan City, South East of Iran, in 2014.

### 2.2. Setting and Samples

There are six teaching hospitals in the study region. The sample was all nurses who were at work during data collection in the hospitals. The participants' verbal informed consent was obtained and nurses participated voluntarily in the study.

The stratified random sampling method was used as follows. Zahedan City was divided into six hospitals (stratum). The nurses' population in each hospital was considered as study population. Then, based on nurses personnel list (sampling frame) a proportional random sample from each hospital was taken by the stratum's size. Considering the proportion of barriers to implementation of EBP to be 78.6% [18], the sample size of 270 at 95% significant levels and error level of 0.05 was calculated and ten additional participants were added to the sample size and the overall sample size reached 280 subjects. Exclusion criteria were nurses who had not been at work during data gathering.

### 2.3. Measurements

We used the questionnaire of barriers to implementation of EBP that has been tested for reliability and validity in a previous study [18]. In this study, Cronbach's coefficient alpha of 0.81 indicated sufficient reliability of all statements in the questionnaire. Demographic information was collected such as age, sex, education, job experience, and employment status.

The questionnaire of barriers to implementation of EBP consists of 27 statements with two aspects (organizational aspect: 18 statements, and individual aspect: 9 statements). In both organizational and individual aspects respondents were asked to score the level to which they agree (scored as 1), have no opinion (scored as 2), or disagree (scored as 3).

### 2.4. Data Analysis

Descriptive statistics (frequency table, mean and standard deviation) were conducted to describe the background factors and barriers to implementation of EBP. The Chi-square test was also used for data analysis at 0.05 significance levels using the statistical software STATA 11 (StataCorp, College Station, TX, USA).

## 3. Results

The questionnaires were distributed to 280 nurses, with 263 nurses returning the questionnaires (response rate = 93.9%).  Table 1 presents the distribution of demographic characteristics of the participants.

The sample included 70.3% women. The average age was 28.4 years (SD = 5.4) with a range of 21–50 years. Most of the participants were employed with education level of Bachelor's degree (94.7%). Most of them (60.5%) had job experience less than 5 years.

More than half of the participants agreed that 56% and 57% of barriers to implementation of evidence based practice are related to organizational and individual aspects, respectively.

The list of five common barriers to implementation of evidence based practice (EBP) by organizational and individual aspects is presented in Table 2. The lack of human resources (shortage of nurse) (78.3%), lack of internet access at work (72.2%), and heavy workload (70.0%) are the most important organizational barriers. The most important individual barriers to implementation of EBP are lack of time to read literature (83.7%), lack of ability to work with computer (68.8%), and insufficient proficiency in English language (62.0%).

Table 3 demonstrates the associations between background variables and barriers to implementation of evidence based practice by organizational and individual aspects. There was a statistical and significant correlation between age, educational level, job experience, employment status, and barriers to implementation of evidence based practice experienced by nurses associated with organizational aspects. With regard to barriers to implementation of evidence based practice experienced by nurses associated with individual aspects, only educational level was significant (*P* = 0.017).

## 4. Discussion

The present study has demonstrated the barriers to implementation of EBP among an Iranian nurses population. The results showed that both organizational and individual aspects are barriers to implementation of EBP.

We found that 57% of barriers to implementation of EBP are related to individual aspects. The three individual barriers most often encountered are lack of time to read literature, lack of ability to work with computer, and insufficient proficiency in English language. This finding is consistent with other studies. The most commonly reported personal barrier is lack of time in different parts of world [19–21]. A qualitative study to explore nurses' experiences and perceptions about EBP showed that over half of participants (52.6%) had not passed any courses on computers [15]. Previous studies [22, 23] revealed that the important factor to find the best evidence to clinical practice questions is having the ability to work with computers. In addition, an Iranian study reported that 21.4% of barriers to implementation of EBP are related to individual aspects. The authors showed that lack of time is a common barrier to implementation of EBP [18]. Similar finding is shown in the other medical groups in the literature. For example, a study to determine the barriers of EBP among Iranian urologists found that being familiar with evidence is needed and lack of time is the important barrier to implementation of EBP [24]. Our study also found that language barriers were another important barrier; insufficient familiarity with English language was a significant barrier to EBP implementation. This is supported by studies conducted in Taiwan showing that nurses preferred evidence based resources to be available in Chinese [20, 25].

Another important finding of this study was that the 56% of barriers to the implementation of EBP were related to individual aspects. The most frequently reported organizational barriers to implementation of EBP were lack of human resources (shortage of nurse), lack of internet access at work, heavy workload, and lack of access to a rich library with nursing journals. Varaei et al. [15] reported that in the human resource category, shortage of nurses and heavy workload are the most common barriers to implementation of EBP. Another study exploring the relationship between nurses' personal and professional factors and EBP found that only 32% of nurses have a library rich in nursing journals at their workplace and 42% had no internet access [13]. Moreover, the results of a study showed that the most important facilitators to the utilization of research in practice are human resources [5]. Accordingly, organizational support can be a target of change in EBP implementation. In this regard, Schoonover [26] concluded that organizational strategies are needed to influence research awareness and utilization. Another study revealed that several problems of clinical nurse performance appraisal system are related to organizational context. Accordingly, changing of the appraisal system is necessary to support the achievement of high quality of patient care [27].

The background variables such as age, educational level, job experience, and employment status were found to be significantly associated with barriers to implementation of EBP. According to the result, participants with job experience less than five and more than 16 years agree more that organizational aspects are associated with barriers to implementation of EBP. In other words, there was a U-shape association between job experience and organizational barriers of EBP implementation. It was identified that nurses with Bachelor's degree agree more that organizational aspects are associated with barriers to implementation of EBP than those with Master's degree. This issue probably is due to small sample size in subgroup of education. Education level has been mentioned in some of the related studies as a main factor to implementation of EBP [13, 28]. Weng et al. [20] also revealed that academic degree and educational training are important factors to implementation of EBP. Since EBP is a critical and important issue in nursing, nurses should increase their knowledge and attitude about EBP and use it for better delivery services. Beside, policy makers must provide a suitable workplace or opportunities for staff to increase their knowledge in hospitals.

In this study older individuals indicated that organizational aspects are important barriers, more than younger individuals. This result probably has some reasons. Older individuals may be more aware of the current trends or know that EBP is something that they should be doing. Another reason is that they are more familiar with hospital system and factors associated with use of EBP. This finding is inconsistent with research by Thompson et al. revealing that demographic variables are not associated with EBP in nurses [29].

### 4.1. Limitations

There are some limitations that should be noted. Self-report questionnaire was used to obtain the data which can introduce information biases. For example, the construction of questionnaire, lack of time, and the individual interest may be derived from self-report questionnaire. Another important limitation of our study was the limited sample size in subgroup of variables such as education level (94.7% versus 5.3%). Despite these limitations, the high response rate (93.9%) could be the strength of this study.

## 5. Conclusions

Barriers to implementation of EBP occur at both individual and organizational levels. The indicator of quality in nursing practice is EBP. Hence, familiarity with EBP is recommended for Iranian nurses. In addition, knowledge of barriers will help health care system and policy makers to address these and to provide a culture of EBP.

## Figures and Tables

**Table 1:** Distribution of participant demographics in Zahedan teaching hospitals.

Variables	Number	%
Age group		
<25 yr	123	46.8
+26 yr	140	53.2
Mean (SD)	28.4 (5.4)	
Gender		
Male	78	29.7
Female	185	70.3
Education		
Bachelor's degree	249	94.7
Master's degree	14	5.3
Job experience (years)		
<5	159	60.5
6–10	62	23.6
11–15	23	8.7
>16	19	7.2
Employment status		
Temporary	185	70.3
Permanent	29	11.1
Fixed term	49	18.6
Barriers to implementation EBP associated with organizational aspects		
Agree	148	56.0
No comment	70	27.0
Disagree	45	17.0
Barriers to implementation EBP associated with individual aspects		
Agree	150	57.0
No comment	58	22.0
Disagree	55	21.0

EBP: evidence based practice.

**Table 2:** The list of five common barriers to implementation of evidence based practice (EBP) by organizational and individual aspects.

	Agree	No comment	Disagree
Organizational aspects			
Lack of human sources	206 (78.3)	44 (16.7)	13 (5.0)
Heavy workload	190 (72.2)	39 (14.8)	34 (13.0)
Lack of access to a rich library with nursing journals	184 (70.0)	57 (21.7)	22 (8.3)
Lack of internet access at work	167 (63.5)	57 (21.7)	39 (14.8)
No cooperation by physicians	163 (62.0)	63 (24.0)	37 (14.0)
Individuals aspects			
Lack of time to read literature	220 (83.7)	28 (10.7)	15 (5.6)
Insufficient proficiency in English language	181 (68.8)	32 (12.2)	50 (19.0)
Lack of ability to work with computer	163 (62.0)	43 (16.3)	57 (21.7)
Lack of autonomy to change practice	145 (55.2)	59 (22.4)	59 (22.4)
Lack of knowledge	143 (54.4)	47 (17.9)	73 (27.7)

Figures are number (%).

**Table 3:** Associations between background variables and implementation of evidence based practice by organizational and individual aspects using Chi-square test.

	Organizational aspects			Individual aspects		
	Agree	Disagree	*P* value	Agree	Disagree	*P* value
Age group (years)						
<25	105 (85.4)	18 (14.6)	0.013^*^	91 (74.0)	32 (26.0)	0.334
>26	102 (72.9)	38 (27.1)		96 (68.6)	44 (31.4)	
Gender						
Male	66 (84.6)	12 (15.4)	0.129	56 (71.8)	22 (28.2)	0.872
Female	141 (76.2)	44 (23.8)		131 (70.8)	54 (29.2)	
Education						
Bachelor's degree	199 (79.9)	50 (20.1)	0.043^*^	181 (72.7)	68 (27.3)	0.017^*^
Master's degree	8 (57.1)	6 (42.9)		6 (42.9)	8 (57.1)	
Job experience (years)						
<5	135 (84.9)	24 (15.1)	0.001^*^	120 (75.5)	39 (24.5)	0.138
6–10	43 (69.4)	19 (30.6)		37 (59.7)	25 (40.3)	
11–15	11 (47.8)	12 (52.8)		16 (69.6)	7 (30.4)	
+16	18 (94.7)	1 (5.3)		14 (71.1)	5 (28.9)	
Employment status						
Contractual	155 (83.8)	30 (16.2)	0.008^*^	134 (72.4)	51 (27.6)	0.122
Permanent	19 (65.5)	10 (34.5)		16 (55.2)	13 (44.8)	
Official	33 (67.4)	16 (32.6)		37 (75.5)	12 (24.5)	

Figures are number (%);  ^*^ *P* value <0.05.
